# Functional Multilayer Biopolymer Films with Botanical Additives for Sustainable Printed Electronics

**DOI:** 10.3390/ma18184328

**Published:** 2025-09-16

**Authors:** Nikola Nowak-Nazarkiewicz, Wiktoria Grzebieniarz, Beata Synkiewicz-Musialska, Lesław Juszczak, Agnieszka Cholewa-Wójcik, Ewelina Jamróz

**Affiliations:** 1 Department of Chemistry, University of Agriculture, Balicka 122, 30-149 Kraków, Polandewelina.jamroz@urk.edu.pl (E.J.); 2 Łukasiewicz Research Network—Institute of Microelectronics and Photonics, Kraków Division, Zabłocie 39, 30-701, Kraków, Poland; 3 Department of Dietetics and Food Studies, Faculty of Science and Technology, Jan Długosz University in Częstochowa, Armii Krajowej 13/15, 42-200 Częstochowa, Poland; 4 Department of Analysis and Evaluation of Food Quality, University of Agriculture, Balicka 122, 30-149 Kraków, Poland; 5 Department of Packaging and Logistics Processes, Cracow University of Economics, Rakowicka 27, 31-510 Kraków, Poland

**Keywords:** biopolymer multilayer films, smart packaging materials, properties of packaging materials

## Abstract

In this study, multilayer biopolymer films composed of furcellaran, chitosan, and gelatin were incorporated with aqueous extracts of *Lavandula angustifolia* and *Clitoria ternatea*. These materials were engineered as sustainable, biodegradable substrates suitable for screen-printing applications. The primary objective was to enhance the films’ functional properties, including their mechanical integrity, barrier performance, and printability, thereby broadening their potential utility in environmentally responsible technological applications. FTIR and UV–Vis analyses confirmed the presence of functional groups associated with the contained plant extracts and showed significantly improved UV-blocking properties. Thermal and mechanical tests showed that the films maintained good structural integrity, and only high extract concentrations slightly affected tensile strength. Importantly, the materials exhibited gradual but limited thermal shrinkage (<3.7%) up to 130 °C, while maintaining their multilayer structure. Water-related evaluations, including WCA, solubility, pH, and conductivity, confirmed their biodegradability in aqueous environments without exceeding ecotoxicological thresholds. Microbiological tests demonstrated selective antimicrobial activity. The key novelty of this work is the evaluation of these active multilayer biopolymer films as screen-printing substrates. This is the first report in which screen-printing compatibility with active multilayer biopolymer systems is presented, highlighting their potential in sustainable packaging that integrates biodegradable matrices with printed sensor layers.

## 1. Introduction

The packaging materials industry remains predominantly reliant on raw materials of petrochemical origin. Due to their non-renewable nature, limited recyclability, and lack of biodegradability, these materials substantially contribute to environmental degradation, particularly in the context of widespread single-use plastic consumption. Due to the origin of the materials, low recycling efficiency, and lack of biodegradability, they leave a strong negative impact on the environment, especially in the context of single-use plastics. Micro- and macro-plastics derived from materials of petrochemical origin degradation are present in soil and water. Thus, they pose a threat to aquatic organisms, soil fertility, and human health. Given this, it has become important to look for materials that can be an alternative to synthetic plastics, and this place has been assumed by biopolymer materials, which are forecast as a good alternative [1,2].

Biopolymers of natural origin, including polysaccharides and proteins, exhibit biodegradability, non-toxicity, and good film-forming properties. However, the functional properties of biopolymer materials, such as solubility, permeability to water vapor and gases, mechanical properties, or UV degradation, often prove insufficient in practical applications. For this reason, increasing attention is being paid to strategies for modifying their properties by adding natural plant extracts and designing multilayer structures. The introduction of aqueous extracts, rich in phenolic compounds, flavonoids, and anthocyanins, enables not only improving the functionality of the material (including antioxidant and antibacterial activity) but also modifying its optical and surface characteristics. In turn, the multilayer architecture enables the selective distribution of active ingredients in specific layers, which optimizes both the protective properties and mechanical durability of the film [3,4].

Accordingly, packaging materials should no longer perform only protective functions; they are increasingly expected to have additional activity (e.g., antimicrobial and antioxidant) and to respond to changing environmental conditions intelligently. Such active and intelligent films can, among others, monitor food quality, inform about freshness, or protect their contents from degradation. In this context, the search for innovative methods of functionalizing biopolymer materials becomes justified. The development of a suitable biopolymer matrix is also important. Furcellaran, which belongs to the group of anionic polysaccharides, is extracted from red algae. This biopolymer exhibits very good film-forming properties, making it a good matrix for the formation of multilayer and active materials. Chitosan, as a product of partial deacetylation of chitin, provides antimicrobial properties in addition to its ability to form stable films. Gelatin, on the other hand, acts as a binding and plasticizing component, favorably influencing the flexibility and processability of the material. The development of multilayer structures using the aforementioned polymers makes it possible to obtain systems with varied functionality and tailored physicochemical properties [3,5,6,7].

One of the innovative solutions to functionalize biopolymer materials may be the use of functional printing technology, such as screen printing, enabling the integration of sensory or conductive layers directly on the surface of biodegradable matrices. To date, research has mainly been focused on homogeneous polymer substrates, while there is a lack of reports on the use of screen printing for materials with multilayer structures and, at the same time, on the potential for active performance [8,9,10]. The present study is an attempt to fill this gap by evaluating the suitability of the developed films as a matrix for printing functional layers, which could be a step toward the development of next-generation biodegradable smart packaging materials. In addition, there are no reports in the literature on the modification of the active properties of biopolymer materials using two flower extracts from *Lavandula* and *Clitoria ternatea*, despite many reports regarding their active effects. Phenolic compounds, containing anthocyanins and flavonoids, present in the mentioned plants, can affect the optical and color properties of the materials and impart antimicrobial or antioxidant properties as well. The use of natural plant additives can also modify the surface and aqueous properties of films, thereby expanding their range of potential applications [11,12,13,14].

The purpose of the present study was to obtain and characterize triple-layer biopolymer films based on furcellaran, chitosan, and gelatin, enriched with aqueous plant extracts (lavender and clitoria, also known as butterfly pea). The examination included evaluation of their physicochemical properties by performing FTIR and SEM analysis. Optical properties were assessed via UV-VIS analysis. Further studied were the opacity degree, film color, and mechanical and thermal properties using DSC. Testing temperature resistance, aqueous properties, permeability to water vapor, water contact angle, solubility, and environmental and microbiological effects were also assessed, as well as suitability as biodegradable substrates for screen-printing of functional layers, with potential application in modern packaging systems.

## 2. Materials and Methods

### 2.1. Materials Used for Preparation of Multilayer Films

Furcellaran (type 7000, Mw 2.951 × 10^5^) was obtained from Est-Agar AS (Karla Village, Estonia). The chemical composition provided by the manufacturer is 79.61% (carbohydrates), 1.18% (protein), and 0.24% (fat). Gelatin derived from porcine skin, glycerol, and 99%acetic acid were obtained from Chemland (Stargard, Poland). Low-molecular-weight chitosan (90% deacetylation) was procured from Pol-Aura (Zabrze, Poland). The flowers of butterfly pea (*Clitoria ternatea* L.) and lavender (*Lavandula* L.) were obtained from Plantago (Złotów, Poland).

### 2.2. Preparation of Triple-Layer Films

Multilayer biopolymer films were prepared using the previously described layer-by-layer method [15,16], in which film-forming gels are prepared so that each layer is capable of forming a gel.

The first layer was formed by depositing a 2% (*w*/*v*) furcellaran solution containing 10% (*v*/*v*) lavender water extract, followed by a similar 2% furcellaran layer supplemented with 10% (*v*/*v*) *Clitoria ternatea* water extract, and capped with a chitosan–gelatin (2:8) complex. Aqueous plant extracts were prepared by mixing an appropriate weight of *Clitoria ternatea* or *Lavandula angustifolia* flowers with distilled water to obtain a 10% (*w*/*v*) ratio. The mixtures were then placed on a magnetic stirrer and heated to 70 °C, maintaining constant stirring until complete extraction was achieved. The chitosan solution (1% *w*/*v*) was prepared in 2% (*v*/*v*) acetic acid, while the gelatin solution was prepared at a concentration of 7% (*w*/*v*). Glycerol was used as a plasticizer. The solutions were mixed using a magnetic stirrer (Heidolph, Kraków, Poland). Each subsequent layer was poured onto the previous one after it had formed a gel. The film-forming solutions were cast onto trays (37 × 29 cm) and dried under a fume hood at ambient temperature. The control film was composed of only the biopolymers.

Three types of films were developed, differing in concentration of the incorporated plant extracts. Based on literature reports, the concentrations of plant extracts (2.5–7.5% *v*/*v*) were selected to provide antimicrobial activity. These levels were chosen to ensure functional effectiveness while not adversely affecting the structural and functional properties of the films [17,18,19]. In the lowest concentration variant (LC-L), lavender and *Clitoria ternatea* extracts were added at 2.5% (*v*/*v*) each; in the medium concentration (LC-M), they were added at 5% (*v*/*v*) each; and in the high concentration variant (LC-H), they were added at 7.5% (*v*/*v*) each relative to the volume of the film-forming solution.

### 2.3. FTIR Spectroscopy

The FTIR spectra of the biopolymer-based films were recorded within the range of 4000–700 cm^−1^ using the Nicolet iS5 spectrometer (ThermoFisher Scientific, Waltham, MA, USA). Due to the specific composition of the multilayer structure, both the first and third layers were subjected to individual analysis.

### 2.4. UV–Vis Spectroscopy Analysis

Spectral analysis in the UV-vis range was performed using the Shimadzu 2101 spectrophotometer (Shimadzu, Kyoto, Japan) at wavelengths ranging from 300 to 700 nm. The opacity measurement was conducted at 600 nm and in accordance with the method described by Du et al. [20]. The results were calculated using the following equation:

(1) $$Opacity=\;A_{600\;}÷\;X$$

where *A*_600_—absorbance value at 600 nm; *X*—film thickness [mm].

The film thickness was measured using a handheld device, Mitotuyo, No. 7327 (Kawasaki, Japan).

### 2.5. Color Parameters

The surface color of the films was determined using the Color i5 spectrophotometer (X-Rite, Grand Rapids, MI, USA) operating in reflectance mode under D65 illumination. The measurements were performed using d/8 geometry, a 10° standard observer, within the spectral range of 400–700 nm, and with a measurement aperture of 25 mm. The color coordinates were expressed in terms of L* (lightness), a* (red–green coordinate), and b* (yellow–blue coordinate). Additionally, the yellowness index (YI), whiteness index (WI), browning index (BI), and total color difference (ΔE) were calculated using the following equations:

(2) $$WI=100-\sqrt{{\left(100-L^{*}\right)}^{2}+{\left(a^{*}\right)}^{2}+{\left(b^{*}\right)}^{2}}$$

(3a) $$BI=100\times \frac{X-0.31}{0.172}$$

(3b) $$X=\frac{a^{*}+1.75L^{*}}{5.645L^{*}+a^{*\;}-3.012b^{*}}$$

(4) $$\Delta E=\sqrt{{\left(L-L^{*}\right)}^{2}+{\left(a-a^{*}\right)}^{2}+{\left(b-b^{*}\right)}^{2}}$$

(5) $$\mathrm{YI}=\frac{142.86\times b^{*}}{L^{*}}$$

where Δ*E* is the total difference in color value between the control films and the films containing extracts from butterfly pea and lavender flowers.

### 2.6. Water Solubility

The solubility of the materials was examined by measuring the conductivity and pH value of the water contained in the samples. The laboratory testing station was prepared, focusing on the sterilization of containers to prevent any potential contamination. Each sample was prepared by combining 1 g of material with 100 mL of distilled water, with three replicates per material to ensure statistical validity. Initial sample weights were measured using a precision scale (RADWAG AS 60/220, Radom, Poland) before complete immersion in water-filled containers. Additionally, the initial pH levels and conductivity of the water were measured using the ELMETRON CPC-411 (Zabrze, Poland) waterproof pH/conductivity meter equipped with a pH sensor (EPS-1, Merazet Poznań, Poland) and a conductivity sensor (ECF-1, Elmetron, Zabrze, Poland) to provide a baseline for future comparison.

Throughout the incubation period, the containers were kept in a temperature-controlled environment (20–23 °C) and sealed to ensure consistent conditions. To capture a broad spectrum of material behavior in time, the incubation periods varied, ranging from 1 to 48 h. At predetermined intervals within these periods, the pH of the water solution after sample removal and the conductivity of the solution were measured using the same ELMETRON CPC-411 (Zabrze, Poland) meter setup to determine the potential influence of the dissolved samples on the environment. The appearance of the film was also analyzed throughout the measurement period.

### 2.7. Water Vapor Transmission Rate (WVTR)

Water vapor permeability of the materials was established according to ISO 2528:2017 [21]. A dish filled with silica gel and covered with the tested materials was placed in a climatic chamber with controlled environmental conditions. The analysis was performed at 75% relative humidity and a temperature of 25 °C.

(6) $$WVTR\;(g÷m^{2}\times d)=240\times (water\;weight\;÷\;surface\;penetration)\times 24$$

### 2.8. Contact Angle Determination

The water contact angles of the materials were determined using the sessile drop method at room temperature. Using a microinjector, a droplet of deionized water (8 μL) was deposited onto the film surface. The contact angle was then measured using a video-based contact angle measurement system (OCA 15EC, Dataphysics, Filderstadt, Germany) equipped with SCA20 Software ver. 5.0.41.

### 2.9. Thermal Properties

Thermal properties of the films were analyzed using a differential scanning calorimeter (DSC 4000, PerkinElmer, Springfield, IL, USA). Samples (approximately 8 mg) were hermetically sealed in aluminum pans and heated from 30 °C to 250 °C at a heating rate of 10 °C/min. An empty aluminum pan was used as the reference. The temperatures and enthalpy changes of thermal transitions were determined using Pyris software ver. 13.3.30032 (PerkinElmer, USA).

### 2.10. Temperature Resistance Tests

For temperature resistance measurements, sample dimensions (X, Y) were measured using a standard caliper before and after temperature treatment (range: 60 to 130 °C, step 10 °C, time 20 min) in a GOLDBRUM 1450 vacuum dryer (Expondo, Zielona Góra, Poland). In the next step, the shrink ratio (%) was calculated for each of the substrates. The behavior of the samples during heating demonstrates how the addition of water extracts from butterfly pea flowers and lavender flowers affects the thermal stability of the samples [22].

### 2.11. Mechanical Properties

Mechanical properties of the materials were determined according to EN ISO 527 [23] and ASTM D882-02 [24] international standards on a bursting machine (Shimadzu EZ test, Kyoto, Japan). Tensile strength (TS), maximum breaking load (MBL), and elongation at break (EAB) were evaluated. The tests were performed at a crosshead speed of 70 mm/min.

### 2.12. Microbiological Analysis

Microbiological activity was evaluated according to the methodology outlined by Grzebieniarz, Tkaczewska, Juszczak, Kawecka, Krzyściak, Nowak, Guzik, Kasprzak, Janik, and Jamróz [17], with slight modifications. The analysis focused on the effect of the films against microorganisms responsible for food spoilage as well as foodborne pathogens, including Gram-positive and Gram-negative bacteria, yeasts, and molds. The analysis was conducted using the following microorganisms: *Candida albicans* ATCC 10231, *Candida krusei* ATCC 6258, *Aspergillus brasiliensis* ATCC 204304, *Aspergillus flavus* ATCC 16404, *Escherichia coli* ATCC 25923, *Enterococcus faecalis* ATCC 29212, *Pseudomonas aeruginosa* ATCC 27869, *Staphylococcus aureus* ATCC 25922, and *Salmonella enterica* BAA664. Petri dishes (⌀90 mm) were employed, containing a solidified Müller–Hinton agar medium for bacteria and Sabouraud agar with glucose for yeasts. A previously prepared bacterial suspension (0.5 on the McFarland scale) was spread on the medium using the lawn method. Then, 0.5 cm diameter film samples were placed on the prepared agar medium in aseptic conditions and incubated at 37 °C for 24 h. The materials were previously sterilized using a UV lamp. Visual analysis was performed to assess the growth of microorganisms in the areas around and above the films.

### 2.13. Assessment of Biopolymer Films as Substrates for Functional Printing

#### 2.13.1. Screen-Printing Tests

The compatibility of substrates with screen-printed (ZUT, Szczecin, Poland) functional layers was verified by realizing test prints using commercial silver-based (DP5000, DuPont, Wilmington, DE, USA) and carbon-based (DP7105, DuPont, USA) paste (dried at 80 °C). The integration of the paste with the substrate material was examined using SEM analysis at various magnifications using the Quattro ESEM microscope (ThermoFisher Scientific) equipped with FEG (field emission gun, ETD, Everhart-Thornley detector).

#### 2.13.2. Microstructure Characterization

The surface of the examined samples, both printed and unprinted, as well as the quality of printed layers, were characterized using the Quattro ESEM microscope (ThermoFisher Scientific) equipped with FEG (field emission gun, ETD, Everhart-Thornley detector). SEM images of sample cross-sections and the surface were obtained using the LVD (low-voltage detector), a mode of magnification ranging from 100 to 1600, and an accelerating voltage of 10 kV.

### 2.14. Statistical Analysis

Statistical analysis of the results was performed using Statistica software (version 13.0, Tibco Software Inc., Palo Alto, CA, USA). One-way analysis of variance (ANOVA) was used, followed by Tukey’s post hoc test. Differences between mean values were considered significant at the level of *p* < 0.05. All experiments were performed at least three times (n ≥ 3), and the results are expressed as mean ± standard deviation (SD).

## 3. Results and Discussion

### 3.1. Fourier Transform Infrared (FTIR) Spectroscopy

**Figure 1:**
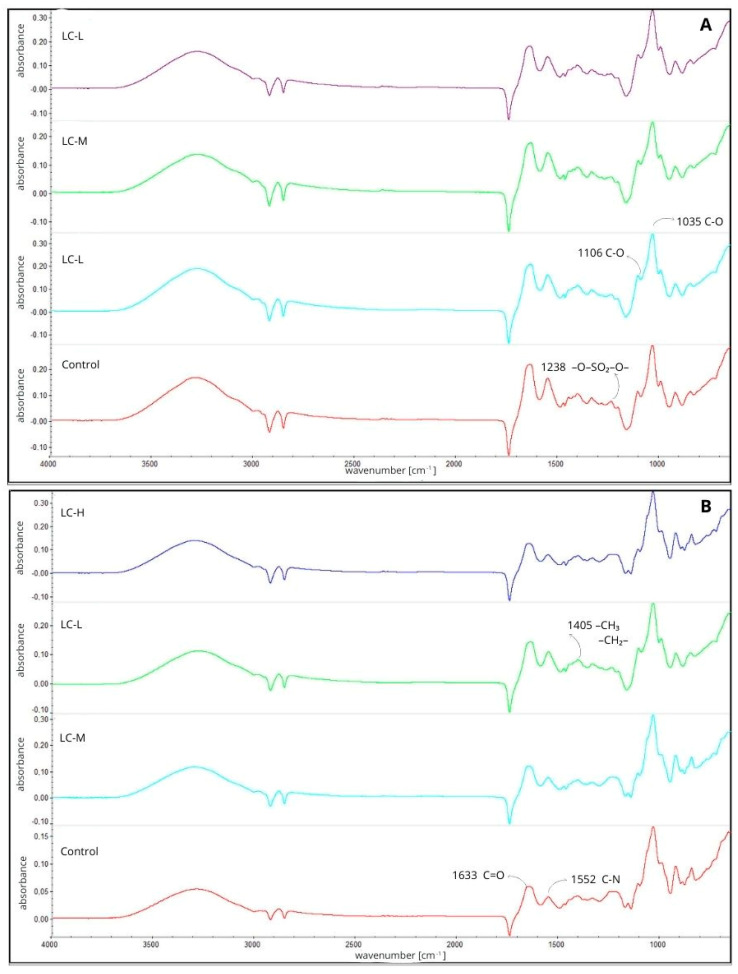
FTIR spectra of the tested materials ((**A**) 1st layer, (**B**) 3rd layer).

Infrared spectral analysis (FTIR) is one of the primary tools used to verify the chemical composition and presence of characteristic functional groups in biopolymer materials. It allows not only to confirm the presence of individual film layer components, but also to assess potential interactions occurring between biopolymers and functional additives, which consequently have a direct impact on film properties. The results of FTIR analyses regarding the analyzed materials are shown in Figure 1.

The FTIR spectra of the tested materials are presented in Figure 1A,B. In Figure 1A, the first layer of furcellaran-based film is shown, where a characteristic band at approximately 1238 cm^−1^ is observed, indicating the presence of sulphate ester groups, which confirms the structure of this polysaccharide. Additionally, signals in the vicinity of 1106 cm^−1^ and 1035 cm^−1^ are visible in the spectrum, which are associated with C–O bridge vibrations and C–O bond stretching [25]. In Figure 1B, the IR spectrum is demonstrated, with bands characteristic of gelatin, corresponding to amide I (approx. 1633 cm^−1^, associated with C=O stretching vibrations) and amide II (approx. 1552 cm^−1^, associated with C–N vibrations) [26]. Peaks within the range of 930–1125 cm^−1^ are attributed to C–O–C bond vibrations characteristic of chitosan. The peak around 1400 cm^−1^ is typical for C–H groups, while the signal at around 1550 cm^−1^ corresponds to NH_2_ amino groups [27].

The spectra also recorded signals that may be associated with the presence of plant extracts. The band in the 1405 cm^−1^ region is attributed to vibrations of methyl and methylene groups, typical of compounds found in lavender, such as linalool and linalyl acetate. The peak around 1336 cm^−1^ indicates the presence of hydroxyl groups characteristic of polyphenols, which naturally occur in plant extracts [12,28].

The analyzed FTIR spectra do not show significant differences between the control samples and those containing the extract, which may be due to the presence of similar functional groups in both biopolymers and plant extracts. However, the absence of pronounced spectral changes does not exclude the occurrence of interactions between the matrix components and the active compounds. These interactions are likely to be non-specific and mainly result from the formation of hydrogen bonds between the hydroxyl groups of polyphenols and the functional groups of biopolymers (–OH, –NH_2_, –C=O). Electrostatic interactions between charged furcellaran groups and protonated amino groups of chitosan are also possible, which may be further stabilized by the presence of extracts. Such bonds and physicochemical interactions may have contributed to the integration of the extracts into the matrix structure without causing any significant shifts in the FTIR spectra, which explains the similarity of the signals in the test and control samples.

### 3.2. UV–Vis Spectroscopy Analysis and Optical Properties

**Figure 2:**
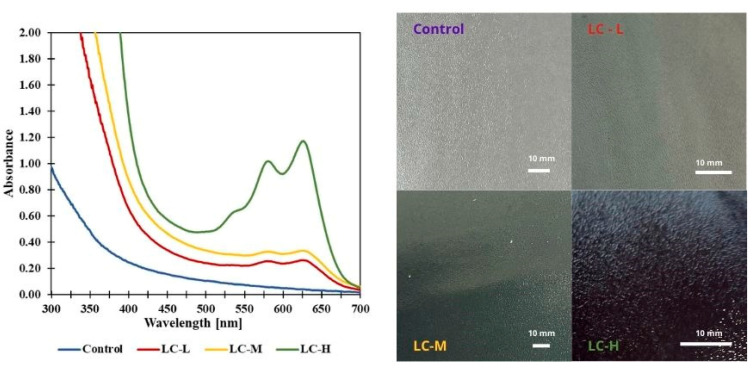
UV–Vis spectra and appearance of films.

The use of ultraviolet–visible spectroscopy allows for determining a material’s ability to block UV radiation and its degree of opacity. Blocking UV radiation is particularly important in the context of the potential use of biopolymer materials as packaging for food products due to their susceptibility to the negative effects of ultraviolet radiation (including lipid oxidation). The UV–Vis spectra of the developed materials are shown in Figure 2.

The optical properties of packaging materials, including both light transmission and color, are also significant from the point of view of consumer perception. The transparency, shade, and overall aesthetics of the material can influence the perception of product freshness and quality [26,29].

The incorporation of plant extracts into the polymer system also allows the optical properties of the film to be modified and gives it functional characteristics. Phenolic compounds, flavonoids, and anthocyanins contained in the extracts can affect the film’s ability to block light and change its color, while also acting as an indicator or protective agent. This makes it possible to obtain active materials—e.g., with antioxidant or antibacterial properties—or smart materials capable of responding to environmental changes. Such properties significantly expand the potential applications of the analyzed systems, especially in the context of sustainable and modern solutions for packaging materials.

UV–Vis spectra (Figure 2) indicate a significant increase in absorbance within the range of 550–650, depending on the concentration of the extract. The highest absorbance values are observed for the LC-H film, while the control film does not exhibit UV-blocking properties. Similar properties for polyvinyl alcohol and chitosan-based films with the clitoria flower extract were also observed by Khanifah et al. [30]. The thickness of the film was also measured, which showed that the thickness of the control film and the LC-L and LC-M samples was in the range of 430–440 µm and did not differ significantly between these variants. In contrast, the thickness of the LC-H film was significantly higher, at 465 µm. This parameter has a direct impact on the optical properties of the material, as the absorbance of UV radiation is proportional to the thickness of the layer through which the light beam passes. Similar relationships have been described in the literature for thin polymer films, where an increase in thickness resulted in higher absorption intensity and changes in the shape of UV–Vis spectra. Compared to the control samples, LC-L, and LC-M, the LC-H film is 6.4%, 8.1%, and 4.7% thicker, respectively. Consequently, the greater thickness of the LC-H film may partially explain the observed differences in its UV barrier properties compared to the other samples, but the concentration of the extract also plays an important role [31,32].

The results are the same for opacity (Table 1). The highest opacity value, and thus the lowest transparency value, was observed in the LC-H sample (2.00), but samples with a lower concentration of active ingredients (LC-L and LC-M) were 5–7 times higher than the control film.

Color analysis (Table 1) showed significant differences in the analyzed parameters due to the presence and volume of the added extracts. The L* brightness parameter is highest in the control sample and decreases with the addition of plant extracts. A similar phenomenon is observed in the case of the yellowness and whiteness indices, which means that with the addition of plant extracts, the films become less yellow and darker. With the addition of plant extracts, a shift in the value of the a* parameter (green-red) toward green, and that of the b* parameter (blue-yellow) toward blue can also be noted. This is consistent with visual observations, opacity values, and the UV–Vis graph. Similar values were obtained by Grzebieniarz, Tkaczewska, Juszczak, Kawecka, Krzyściak, Nowak, Guzik, Kasprzak, Janik and Jamróz [17], and this was due to the addition of clitoria extract with an intense blue-violet color.

### 3.3. Mechanical and Thermal Properties

In addition to the permeability of materials to water vapor and gases, the mechanical properties of materials are considered to be one of the most important performance parameters of materials. The thermal and mechanical properties of the materials are shown in Table 2. In the present study, the incorporation of plant extracts slightly reduced the maximum breaking load and tensile strength values, with the effect being dependent on extract concentration. In contrast, no significant differences were observed between the control sample and the extract-containing films with regard to elongation at break.

Notably, no statistically significant differences in any of the tested mechanical parameters were found between the control and the LC-L and LC-M samples, suggesting that only a high concentration of the extract (LC-H, 15% *v*/*v*) negatively affects the mechanical strength of multilayer films. These findings are consistent with those achieved in a previous work by Grzebieniarz, Tkaczewska, Juszczak, Kawecka, Krzyściak, Nowak, Guzik, Kasprzak, Janik and Jamróz [17], who reported that only low concentrations (5% *v*/*v*) of the *Clitoria ternatea* extract did not impair the mechanical performance of double-layer furcellaran-based films.

The observed mechanical performance may also be influenced by the casting and drying conditions, which determine the internal stress relaxation and surface smoothness of the films, which are directly related to tensile strength and breaking load. In this context, the slightly lower MBL values observed for LC-H may not only result from the higher concentration of extracts, but also from their influence on the arrangement of polymer chains during solvent evaporation.

It is also worth emphasizing that the mechanical parameters obtained in this study—particularly the maximum breaking load—are superior to those reported for other comparable biopolymer systems. The control film in the present work reached a maximum breaking load of 44.44 N, whereas previously developed double-layer furcellaran-based films reached only 26.54 N [17]. Even lower values were observed in triple-layer systems, in which the first two layers were furcellaran and the third was a furcellaran-gelatin complex, with a maximum breaking load of only 15.38 N [26]. This suggests that the specific multilayer configuration developed in this study may positively contribute to the structural integrity and mechanical performance of the films. Subsequently, Alasalvar et al. [33] also observed a deterioration in mechanical properties as a result of adding lavender extract, concluding that polyphenolic compounds may affect the action of plasticizers inside the film.

Analysis of thermal properties (Table 1) showed no significant differences in peak temperature values. The peak melting temperature (Tm) values for all tested samples are within a similar range: from 153.06 ± 3.39 °C to 158.86 ± 3.25 °C. Therefore, the addition of plant extracts did not cause any interactions significantly affecting the thermal stability of the materials [16,34].

However, an increase in the enthalpy change value from 200 (J/g) to 217 (J/g) was observed for LC-H. This may indicate that the addition of natural extracts increases the ability of the materials to form a crystalline structure, and may also indicate a greater proportion of the ordered phase or additional interactions between the components, leading to a more compact film structure. This may have a positive effect on performance properties such as thermal resistance and storage stability.

**Figure 3:**
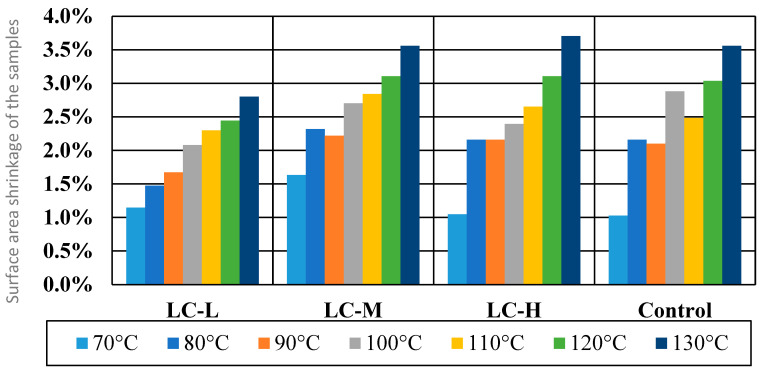
Surface area shrinkage of the samples [%] after thermal treatment (15 min) at 70–130 °C.

In Figure 3, the results are shown for the temperature resistance test in the case of Surface Area Reduction (XY dimensions). All of the samples exhibited a progressive decrease in surface area, indicative of thermal shrinkage. This is consistent with the thermomechanical behavior of polymer matrices undergoing softening, partial melting, or physical cross-linking. The LC-L sample demonstrated the most stable behavior at 130 °C, with the surface area reducing slightly from 25 cm^2^ to 24.30 cm^2^ (≈2.8%). The shrinkage of all samples proceeded gradually, exhibiting relatively good thermal dimensional stability in the planar direction.

Unlike the planar contraction, the thickness behavior (Z-Dimension) of the films was non-linear and more complex, influenced by competing mechanisms such as plasticization, moisture loss, relaxation of internal stresses, and potential microstructural rearrangements. Shrinkage by several percent in the Z axis was observed already at 70 °C for all samples. At temperatures of 100 °C and above, the control sample showed the lowest level of shrinkage but also demonstrated more complex behavior according to characteristics noted in lower temperatures.

The results of the temperature resistance test correspond well with the analysis of thermal (DSC) and mechanical properties. All tested films showed similar melting temperatures (Tm) ranging from 153 to 159 °C, confirming that in the test conditions (up to 130 °C), there was no degradation or melting of the material, only partial softening. The observed linear and moderate surface shrinkage is therefore consistent with the typical behavior of biopolymer matrices in the transition phase, below their melting temperature.

The smallest surface shrinkage of the LC-L sample may be related to its highest melting enthalpy (ΔHm = 217.74 J/g), which indicates a more ordered internal structure and greater resistance to thermal deformation. In contrast, samples with lower ΔHm values showed slightly more pronounced dimensional changes in thermal conditions, which may suggest a lower packing density of the solid phase [35,36].

These observations also partially coincide with the results of mechanical tests—sample LC-L had the highest values of maximum breaking load (MBL) and tensile strength (TS), which may confirm that a more stable thermal structure also translates into greater mechanical resistance in static conditions.

The behavior of the film thickness (Z-dimension) indicates more complex mechanisms of the material’s response to temperature. The observed changes may be the result of competing phenomena, such as moisture loss, thermal swelling, internal stress relaxation, or local structural changes. At lower temperatures (70–90 °C), some samples showed heterogeneous changes in thickness, which may be due to the residual presence of water that underwent evaporation or migration [36,37].

### 3.4. Water Properties and Environmental Impact

As previously indicated, the permeability of materials to water vapor and gases is one of the key performance parameters, particularly since it is with this that biopolymer packaging materials differ significantly from their synthetic counterparts. The aqueous properties of these materials are therefore significant, specifically in the context of their potential use as packaging materials. These properties are shown in Table 3.

Water contact angle (Table 3) analysis showed that the addition of an aqueous lavender flower extract to the first furcellaran-based layer increased the WCA value from 89.73 ± 1.68° for the control sample to 101.08 ± 1.27° for LC-H. This suggests that the incorporation of the aqueous extract contributed to a more hydrophobic surface character, which is extremely important in the context of packaging, especially for food [38].

In the case of the third layer, composed of a chitosan–gelatin complex, a slight decrease in WCA values was observed in samples containing the extract. However, for all variants, values remained below 90°, indicating an overall hydrophilic nature of this surface [38]. No clear dependence on extract concentration was noted. The statistically significant increase in WCA values may be attributed to the presence of hydrophilic phenolic compounds migrating from adjacent layers or to the influence of the extracts on the surface arrangement of polymer components during film formation, as the extracts were not directly present in the third layer [39].

**Figure 4:**
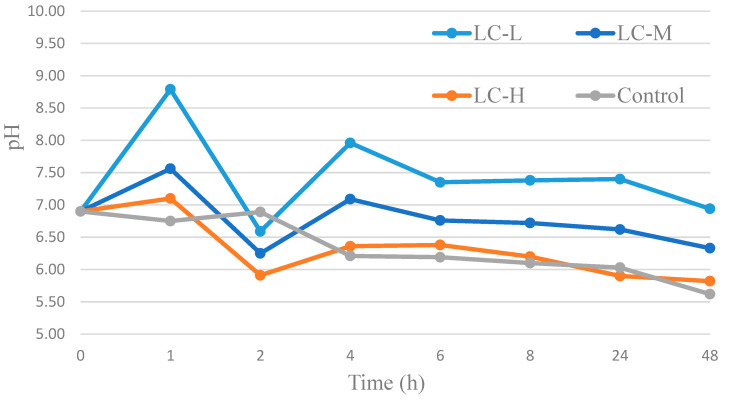
Changes in pH of water containing samples with plant extracts and control sample, measured in time (1–48 h).

Water vapor transmission rate (Table 3) values did not differ significantly among samples and remained within the range of 504–569 g/m^2^·24 h. These findings indicate that the addition of aqueous extracts did not adversely affect the vapor barrier properties of the films, and the multilayer structure remained uniform and structurally intact [40,41,42]. The pH measurements (Figure 4) over a 48 h period provide an understanding of how the materials may influence soil acidity and nutrient availability.

Starting from a neutral pH of 6.90, sample LC-L showed a short-term alkalization (pH 8.79 at 1 h), returning to near-neutral (6.94), suggesting a transient effect likely not harmful to the environment. Samples LC-M, LC-H, and the control decreased gradually to pH 6.33, 5.82, and 5.62, accordingly representing acidification within the tolerable limits for the majority of crops [43]. The tests show limited potential to disrupt nutrient uptake in real field conditions, where locally limited dilution and buffering effects are present. The strongest change in pH was observed during the first 6 h of soaking, which can be associated with the phase of intense swelling and leaching of film components. The initial increase in pH is most likely due to the release of free amino groups of chitosan (−NH_2_ with partial deprotonation) and gelatin components, which may contribute to alkalization of the environment. The subsequent decrease in pH is attributed to the presence of residual acetic acid used to dissolve chitosan and the gradual release of sage extract components, primarily polyphenols (such as rosmarinic acid) and anthocyanins (from *Clitoria ternatea*), whose ionization depends on pH and may affect pH stabilization. The greatest pH variability in the LC-L variant is probably due to the low extract load, which limits the ability to stabilize the pH compared to LC-M and LC-H, where higher concentrations of bioactive components can provide greater stability [44,45,46,47].

The pH range from 6.0 to 7.5 is considered the most favorable for plant growth and is optimal in terms of nutrient availability. Within this range, essential macro- and micro-nutrients are readily accessible to plants, supporting healthy development and crop productivity. Additionally, the broader pH range between 5.0 and 8.0 is beneficial for the activity of soil microorganisms, which play a key role in processes such as mineralization, nitrification, and the decomposition of organic matter. These biological functions are critical for maintaining soil fertility and nutrient cycling. Therefore, these pH ranges are recognized as the most conducive to soil health and maintaining overall quality of the soil environment [22,43].

**Figure 5:**
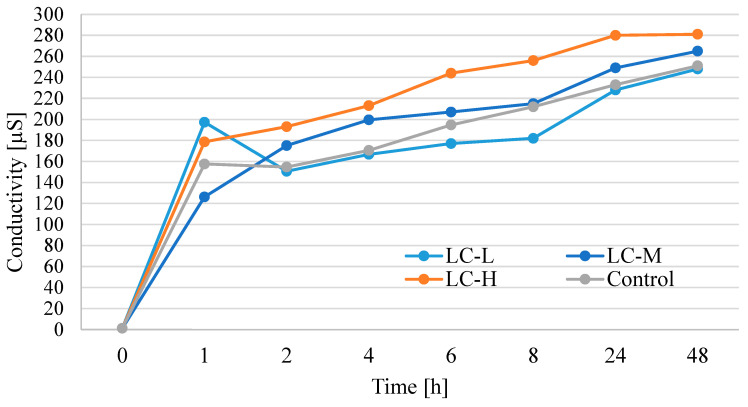
Changes in electrical conductivity among µS in samples containing 1 g of material (LC-L, LC-M, LC-H, and control) with 100 mL of water, measured in time (1–48 h).

In Figure 5, the results are shown for electrical conductivity measurements taken of the mixture containing 1 g of the analyzed films and 100 mL of water, measured at time intervals over a 48 h period. Electrical conductivity reflects the presence of ions leached from materials into water, which may be relevant to soil quality and microbial activity. The baseline conductivity increased significantly across all samples (up to 248–281 µS, dependent on sample). All final conductivity values remained well below levels that could impair soil function (typically 0.6–5 dS/m = 600–5000 µS). Therefore, even the highest measured value (~0.28 dS/m) poses a negligible risk to soil health [43].

In order to assess the solubility of the developed materials in an aqueous environment, a 48 h stability test was conducted, consisting of observing visual changes in samples immersed in distilled water. During the test, three characteristic stages of degradation were identified: waterlogging, delamination, and defragmentation. The results are presented in Table 4.

All analyzed films were quickly waterlogged after just 1 h of contact with the water, followed by delamination and final structural destruction, depending on the formulation. Fragmentation of the material was observed in all samples after 8 h at the latest, and this condition persisted until the end of the observation period (48 h), confirming the complete susceptibility of the materials to water degradation.

Despite the presence of 1% chitosan in the outer layer of the composite (dissolved in a 2% acetic acid solution), no inhibition of the material degradation process was observed. This may be due to incomplete cross-linking of chitosan in an acidic environment or to the overall predominance of hydrophilic components in the film structure. Another reason for these observations may be the simultaneous measurement of pH in the sample, which contributed to defragmentation of the material. Process parameters such as drying temperature and extraction time also affect the aqueous stability of the films. Faster solvent evaporation can increase the porosity of the material, accelerating water uptake and degradation during solubility tests.

### 3.5. Microbiological Analysis

Biopolymer materials show a wide range of applications as smart materials, i.e., those that can perform functions related to packaging as an indicator of product freshness, and as active materials, i.e., those that can influence the freshness of the packaged product [48]. There are several reports in the literature in which the antimicrobial properties of extracts from Clitoria flowers and lavender are outlined, as well as chitosan itself [17,49,50]. The results of the antimicrobial analyses for the obtained composites are given in Table 5.

The results of microbiological analysis indicate that the film has no effect on fungi (*Candida albicans* and *Candida krusei*), molds (*Aspergillus brasiliensis* and *Aspergillus flavus*), or Gram-negative bacteria (*Pseudomonas aeruginosa* and *Salmonella enterica*). However, microbiological analysis showed antimicrobial activity against certain Gram-positive bacteria, with the highest activity against *Staphylococcus aureus* and *Enterococcus faecalis*. Despite numerous reports in the literature on the antimicrobial activity of lavender and butterfly pea flower extracts [51,52], no differences in the antimicrobial activity of the film were observed in vitro as a result of the addition of plant extracts. Perhaps this was caused by the concentration of active ingredients being too low or their very good integration into the matrix, which slowed down the release of the compounds. The antimicrobial activity of the film was most likely due to the presence of chitosan, which has bactericidal properties. The mechanism of chitosan’s antimicrobial activity is mainly related to its effect on the cell membrane of bacteria, disrupting its structure and functions, as well as inhibiting the synthesis of proteins and nucleic acids, which ultimately leads to the death of the bacterial cell [53,54,55].

### 3.6. Assessment of Biopolymer Films as Substrates for Functional Printing

In order to evaluate the potential applicability of the developed biopolymer films as biodegradable substrates for printed electronics, screen-printing tests were conducted using conductive commercial pastes (DuPont DP5000 and DuPont DP7105). The analysis was aimed at determining the compatibility of the material with the printing process and evaluating the quality of adhesion of the functional layer to the film surface. The application of this approach may find practical use in the development of smart, sustainable packaging materials with additional diagnostic or monitoring functions.

**Figure 6:**
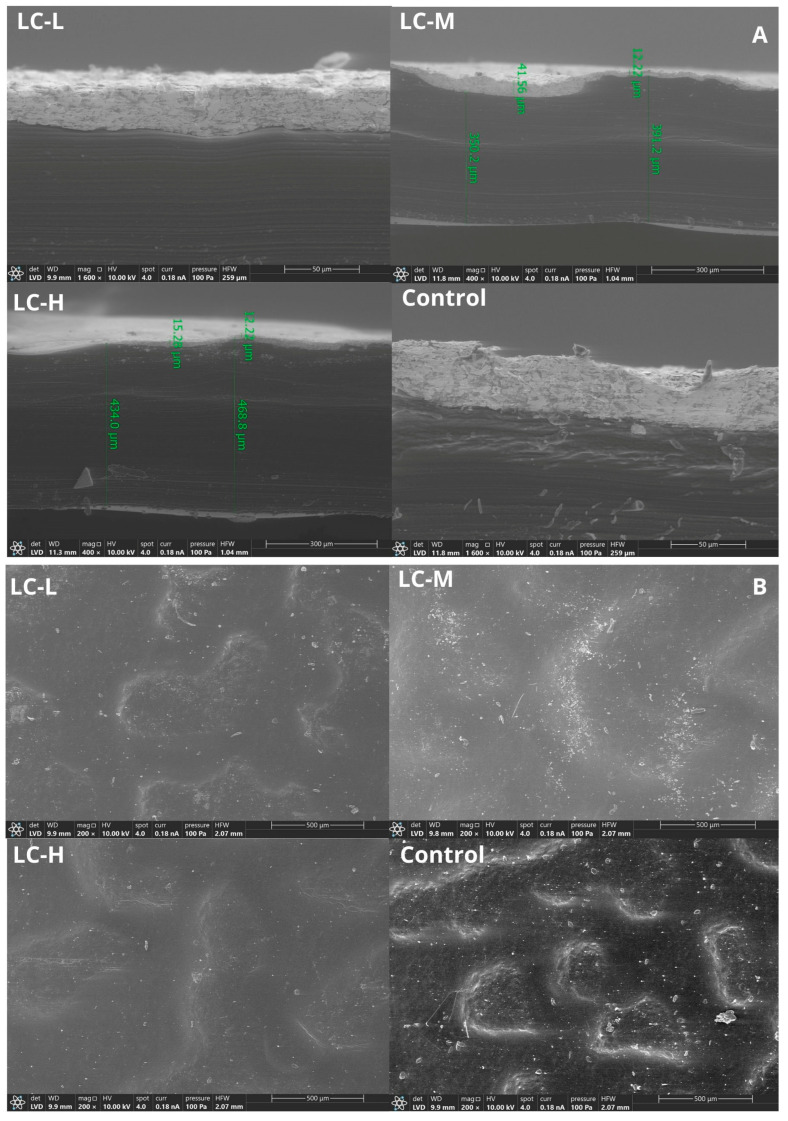
SEM image showing integration of printed layer with substrate (**A**), sample surfaces (**B**), test prints of patterns and electrodes performed using screen-printing method, as well as DP5000 (silver color of print) and DP7105 pastes (black color of print).

**Figure 7:**
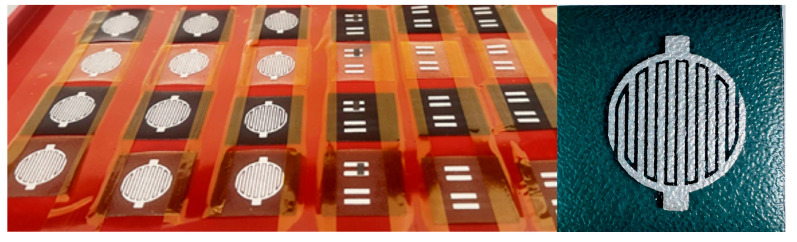
Examples of screen-printed pattern on surface of biopolymer films.

Analysis performed using scanning electron microscopy (SEM) enabled the assessment of both the quality of the printed layer integration and the surface microstructure of the developed multilayer biopolymer materials. The results are presented in Figure 6 and Figure 7. No delamination or cracks in the conductive layers were observed on any of the analyzed samples (LC-L, LC-M, LC-H, and control) after the screen-printing process, indicating good adhesion of the paste to the biopolymer substrate.

The conductive pastes effectively filled the surface irregularities, suggesting good wettability and mechanical anchoring to the material. Nevertheless, due to the characteristic roughness of the film surface resulting from the casting method and the properties of the biopolymers used, the printed layers showed varied topography. This surface heterogeneity may affect the quality of the print, including its conductivity and resolution. Therefore, in order to improve surface homogeneity and print quality, it is recommended to use local thermal pressing before the printing process.

Importantly, cross-sectional analysis also revealed a distinct triple-layer structure of the material, confirming that the layer arrangement retained its mechanical and structural integrity after printing and drying. The clear boundaries between the furcellaran layers and the top layer of gelatin, as well as the chitosan complex, indicate the structural stability of the system.

To summarize the results obtained, it should be noted that the developed multilayer films were characterized by mechanical and thermal stability comparable to or higher than other biopolymer systems described in the literature. The highest breaking strength value (MBL = 44.44 N for the control sample) was significantly higher than for previously developed two-layer films based on furcellaran (26.54 N) [17] or three-layer films with a furcellaran/gelatin complex (15.38 N) [26]. The results obtained suggest that the designed layer architecture, with the separation of plant extracts in the outer layers and the stabilization of the top layer with a chitosan–gelatin complex, promotes the maintenance of the mechanical integrity of the material. At the same time, the observed lack of significant changes in melting temperature (Tm 153–159 °C) confirms that the introduction of natural additives did not disturb the thermal stability of the matrix, which is an important advantage in the context of further processing.

When considering the possibility of scaling up the technology, it is important to emphasise the simplicity of the process used, the preparation of aqueous solutions of biopolymers and extracts, and their application via layer casting. This process is compatible with industrial methods such as solvent evaporation and extrusion, provided that the drying parameters and layer thickness control are optimized [3,56]. This is particularly important to smooth the surface, which could be achieved, for example, by thermal pressing to increase the homogeneity of the layer and improve the conditions for functional printing.

From an economic point of view, this technology is based on readily available raw materials: furcellaran, chitosan, and gelatin are biopolymers widely used in the food and pharmaceutical industries, with relatively low unit costs [57,58]. Plant extracts are a component that could potentially increase costs, but their share in film-forming solutions is low (2.5–7.5% *v*/*v*), which limits their impact on the final price of the product. It is worth noting that the introduction of active functions (antioxidant and antibacterial) using extracts may allow for the elimination of more expensive synthetic additives, which, overall, may increase the competitiveness of the developed system. An additional aspect is the possibility of integrating conductive layers using screen printing, eliminating the need for additional carrier films and lamination processes, which translates into a shorter production chain and reduced costs.

In comparison with other approaches, it is worth noting that films with the addition of nanomaterials, such as nanocellulose, nanoclays, or nanometals, often offer better barrier properties, but raise concerns about safety and legal regulations [59]. Similarly, systems with essential oils exhibit high antimicrobial activity [39,60], but their rapid volatilization and the difficulty in ensuring homogeneity in the polymer matrix may be problematic. Compared to these solutions, the films obtained in this study combine biodegradability, non-toxicity, and the activity of plant extracts with the possibility of further functionalization via sensor printing. Importantly, this is the first report in the literature confirming the possibility of direct application of conductive layers onto active multilayer films with the addition of plant extracts.

In summary, the results obtained show that the developed films can be a viable alternative to currently used active and smart systems, combining favorable mechanical and barrier properties with biodegradability and the possibility of integration with functional printing technologies. This opens up new avenues of research into biopolymers as substrates for printed electronics and applications in next-generation smart packaging systems.

## 4. Conclusions

The obtained results confirm the successful development of multilayer biopolymer films with functional properties tailored through the incorporation of plant-based extracts. FTIR and UV–Vis analyses allowed us to verify the presence of key functional groups and demonstrated improved UV-blocking properties as well as color modulation resulting from the addition of lavender and *Clitoria ternatea* extracts. Thermal and mechanical characterization showed that the materials retained structural stability and performance, with only high extract concentrations slightly affecting strength parameters. All samples showed minor but progressive planar shrinkage (~1–3.7%) over the temperature range up to 130 °C. The vertical dimension displayed more complex thermal responses, driven by the interplay of softening, drying, structural rearrangement, and possibly partial melting or foaming effects. The LC-H sample was particularly sensitive to heat, which was most likely due to the highest concentration of aqueous extracts.

Water-related properties, including water contact angle, solubility, pH, and conductivity, confirmed the films’ biodegradability in aqueous environments and their minimal environmental impact. Microbiological evaluation showed selective antimicrobial activity, mainly attributed to the presence of chitosan.

Finally, screen-printing tests validated the potential of the developed films as substrates for functional layers. The good adhesion and structural stability of the multilayer system after printing suggest practical applicability in the development of intelligent and active biodegradable packaging capable of integrating printed sensors or indicators.

## Figures and Tables

**Table 1:** Optical properties of obtained films.

	Types of Triple-Layer Films			
	Control	LC-L	LC-M	LC-H
Color Parameters				
L*	88.05 ± 0.12 ^d^	58.45 ± 0.57 ^b^	62.45 ± 0.36 ^c^	36.37 ± 1.50 ^a^
a*	−0.26 ± 0.08 ^d^	−13.98 ± 0.22 ^c^	−11.90 ± 0.30 ^b^	−11.31 ± 0.39 ^a^
b*	16.99 ± 0.07 ^d^	6.34 ± 0.46 ^b^	12.53 ± 0.81 ^c^	−3.44 ± 0.35 ^a^
ΔE	-	34.32	28.48	56.67
WI	79.23	55.70	58.67	35.28
YI	27.56	15.50	28.66	−13.53
BI	20.47	−7.29	6.70	−31.35
Opacity	0.11 ± 0.01 ^a^	0.51 ± 0.02 ^b^	0.67 ± 0.04 ^c^	2.00 ± 0.03 ^d^

^a,b,c,d^ different letters indicate significant differences between the measures.

**Table 2:** Mechanical and thermal properties of obtained films.

	Types of Triple-Layer Films			
	Control	LC-L	LC-M	LC-H
Mechanical Properties				
Max. breaking load (MBL) [N]	33.93 ± 1.24 ^a^	31.06 ± 2.03 ^ab^	31.27 ± 0.69 ^ab^	30.20 ± 0.46 ^b^
Tensile strength (TS) [kN/m^2^]	2.26 ± 0.08 ^a^	2.07 ± 0.13 ^ab^	2.08 ± 0.05 ^ab^	2.01 ± 0.03 ^b^
Elongation at break (EAB) [%]	44.44 ± 8.14 ^a^	41.65 ± 2.76 ^a^	41.06 ± 2.19 ^a^	43.53 ± 5.01 ^a^
Thermal Properties				
Peak temperature (Tm) (°C)	156.31 ± 2.42 ^a^	153.06 ± 3.39 ^a^	153.17 ± 3.24 ^a^	158.86 ± 3.25 ^a^
Enthalpy (ΔHm) (J/g)	200.60 ± 7.00 ^b^	214.02 ± 1.50 ^a^	215.17 ± 2.58 ^a^	217.74 ± 3.58 ^a^

^a,b^ different letters indicate significant differences between the measures.

**Table 3:** Water contact angle and WVTR of films.

Types of Triple-Layer Films							
Control		LC-L		LC-M		LC-H	
Water Properties							
Water contact angle [°]	3rd layer (CHIT/GEL)						
	73.06 ± 0.73 ^a^		69.38 ± 4.29 ^b^		69.68 ± 1.15 ^b^		69.09 ± 2.32 ^b^
	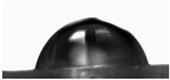		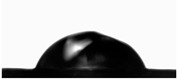		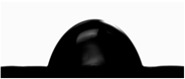		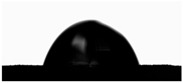
	1st layer (FUR + lavender extract)						
	89.73 ± 1.68 ^a^		106.15 ± 0.68 ^c^		99.99 ± 0.86 ^b^		101.08 ± 1.27 ^b^
	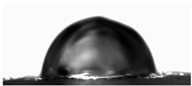		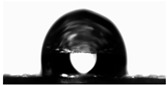		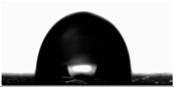		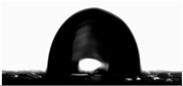
WVTR	504.35 ± 26.74 ^a^		541.47 ± 104.48 ^a^		569.12 ± 63.44 ^a^		539.37 ± 15.99 ^a^

^a,b,c^ different letters indicate significant differences between the measures.

**Table 4:** Appearance of film during solubility tests.

	Time	0 h	1 h	2 h	4 h	6 h	8 h	24 h	48 h
Sample									
LC-L		Waterlogged	Waterlogged	Waterlogged, delamination	Waterlogged, delamination	Waterlogged, delamination	Waterlogged, delamination, defragmentation	Waterlogged, delamination, defragmentation	Waterlogged, delamination, defragmentation
LC-M		Waterlogged	Waterlogged	Waterlogged, delamination	Waterlogged, delamination, defragmentation	Waterlogged, delamination, defragmentation	Waterlogged, delamination, defragmentation	Waterlogged, delamination, defragmentation	Waterlogged, delamination, defragmentation
LC-H		Waterlogged	Waterlogged	Waterlogged, delamination	Waterlogged, delamination	Waterlogged, delamination, defragmentation	Waterlogged, delamination, defragmentation	Waterlogged, delamination, defragmentation	Waterlogged, delamination, defragmentation
Control		Waterlogged	Waterlogged	Waterlogged, delamination	Waterlogged, delamination	Waterlogged, delamination, defragmentation	Waterlogged, delamination, defragmentation	Waterlogged, delamination, defragmentation	Waterlogged, delamination, defragmentation

**Table 5:** Zone [mm] of microbial growth inhibition for analyzed films.

Microorganism	Control	LC-L	LC-M	LC-H
*Candida albicans*	No effect	No effect	No effect	No effect
*Candida krusei*	No effect	No effect	No effect	No effect
*Aspergillus brasiliensis*	No effect	No effect	No effect	No effect
*Aspergillus flavus*	No effect	No effect	No effect	No effect
*Escherichia coli*	4 ± 6.9	12 ± 0.0	14 ± 1.7	9.3 ± 8.0
*Enterococcus faecalis*	10 ± 8.7	14 ± 1.0	15 ± 1.0	14.3 ± 1.10
*Pseudomonas aeruginosa*	No effect	No effect	No effect	No effect
*Staphylococcus aureus*	17 ± 2.0	14 ± 1.0	16.2 ± 0.60	15 ± 1.70
*Salmonella enterica*	No effect	No effect	No effect	No effect

## Data Availability

The original contributions presented in this study are included in the article. Further inquiries can be directed to the corresponding author.
